# Temperature Hysteresis Mechanism and Compensation of Quartz Flexible Accelerometer in Aerial Inertial Navigation System

**DOI:** 10.3390/s21010294

**Published:** 2021-01-04

**Authors:** Chunxi Zhang, Xin Wang, Lailiang Song, Longjun Ran

**Affiliations:** School of Instrumentation and Optoelectronic Engineering, Beihang University, Beijing 100191, China; zhangchunxi@buaa.edu.cn (C.Z.); Vong_Hsin@163.com (X.W.); songlailiang@buaa.edu.cn (L.S.)

**Keywords:** temperature compensation, hysteresis, quartz flexible accelerometer, aerial inertial navigation system, thermal effect, creep effect

## Abstract

Strap-down inertial navigation systems (INSs) with quartz flexible accelerometers (QFAs) are widely used in many conditions, particularly in aerial vehicles. Temperature is one of the significant issues impacting the performance of INS. The variation and the gradient of temperature are complex under aerial conditions, which severely degrades the navigation performance of INS. Previous work has indicated that parts of navigation errors could be restrained by simple temperature compensation of QFA. However, the temperature hysteresis of the accelerometer is seldom considered in INS. In this paper, the temperature hysteresis mechanism of QFA and the compensation method would be analyzed. Based on the fundamental model, a comprehensive temperature hysteresis model is proposed and the parameters in this model were derived through a temperature cycling test. Furthermore, the comparative experiments in the laboratory were executed to refine the temperature hysteresis model and to verify the effectiveness of the new compensation method. Applying the temperature hysteresis compensation in flight condition, the result shows that the position error (CEP) is restrained from 1.54 nmile/h to 1.29 nmile/h. The proposed temperature hysteresis compensation method improves the performance of INS effectively and feasibly, which could be promoted to other applications of INS in similar temperature changing environment correspondingly.

## 1. Introduction

INS is one of the most important measurements in the aerial vehicles. It provides the attitude information to the control system, which can guide an aerial vehicle without external information, such as GPS. Therefore, the performance of INS is vital to aircraft. With the rapid development of inertial technology, the performance of gyroscopes has improved tremendously. Nevertheless, the performance of QFA is not comparable with that of gyroscopes. In an aerial platform, vibration and temperature issues are two of the main factors degrading the performance of the QFA [1,2,3,4].

The QA-3000 manufactured by Honeywell (Morristown, NJ., USA) and the GJN096 manufactured by China Aerospace Science and Industry Corporation (Beijing, China) whose performances are similar cover the medium and high classes of INS. The performances of these two QFAs are shown in Table 1. The thermal issue is the main factor degrading the performance of QFA. Taking the Honeywell QA-3000-030 as an example, the temperature coefficient of bias is typical 15 μg/°C and the temperature coefficient of scale factor is typical 120 ppm/°C, which means when temperature varies from −20 °C to +70 °C, the bias of QFA will drift for nearly 1.35 mg and the scale factor will drift for 10,800 ppm at most, if there is no effective temperature compensation for QFA. Therefore, improving the temperature adaptability of QFA is vital for INS.

Lots of work has been conducted to improve the temperature experiment performance of QFA. Temperature is used to build a general temperature model through simple linear regression and the result shows that the general performance is improved in pure inertial navigation [5,6]. Establishing the temperature model by linear regression is a common strategy. However, the temperature model built by simple linear regression only slightly improves performance [7,8]. In order to optimize the temperature model, an improved linear regression algorithm that focuses on determining the order of the model is proposed. The simulation result indicates that the temperature performance of compensated bias is better compared with a simple regression algorithm [9]. Nevertheless, considering the high performance of INS, these simple linear regression models which only considers thermal effect do not meet the demand for temperature performance. Consequently, a temperature-dependent model based on a neural network is proposed. The drifts of bias and scale factor are well compensated in a wide temperature range [10,11,12]. But the repeatability of this temperature compensation method remains suspicious.

The proposed temperature hysteresis compensation method in this paper is motivated by studying the viscoelasticity of a macromolecular compound in QFA, and it might be a new avenue to support the improvement of the INS. The main contributions of this paper are summarized as follows:

(1) Based on the analysis of the viscoelasticity of epoxy resin (ER), the viscoelasticity of ER is attributed to the main inner factor of bias-hysteresis phenomenon. Multiple piecewise function is applied to bias-hysteresis model dealing with creep whose influence on deformation of ER is irregular.
(2) The temperature hysteresis of magnetic induction is mainly determined by the temperature and temperature gradient, which is the main inner factor of scale factor hysteresis. Therefore, temperature, temperature gradient and the coupling of temperature and temperature gradient are used to build the scale factor-hysteresis model.
(3) Four-points rotation calibration experiments for QFA is used to build the rough temperature hysteresis model. Furthermore, the rough temperature hysteresis model is refined through system-level temperature experiments.

Considering the analysis of the relevant literature, few studies involving the temperature hysteresis of QFA are conducted and its impact on navigation accuracy remains unclear. In this paper, a novel compensation method is proposed to address the temperature hysteresis issue. The rest of this paper is organized as follows: In Section 2, the temperature hysteresis of bias and scale factor will be mainly analyzed. Hysteresis of magnetic conduction is mainly influenced by temperature and temperature gradient, which is the inner factor of scale factor hysteresis. Based on this fact, the temperature hysteresis model scale factor is built. Through ER temperature experiments the viscoelasticity of the ER is attributed to the main factor of bias-hysteresis. In Section 3, a four-point rotation calibration test is designed for three cases of QFAs to build the rough hysteresis temperature model in the laboratory. Furthermore, this rough model is refined by this system-level temperature calibration experiment to form an explicit model which then is applied to the flight condition. In Section 4, the summary of this paper is concluded.

## 2. Analysis of Temperature Hysteresis

### 2.1. Temperature Model of QFA

The common model of QFA is [13]:

(1) $$\begin{array}{ll} E_{out}= & K_{1}K_{0}+K_{1}a_{i}+K_{1}K_{2}a_{i}^{2}+K_{1}K_{3}a_{i}^{3}+K_{1}K_{ip}a_{i}a_{p}\\ & +K_{1}K_{io}a_{i}a_{o}+K_{1}\delta_{o}a_{p}+(-K_{1}\delta_{p})a_{o} \end{array}$$

where $E_{out}$ is the output of QFA; $a_{i},\;a_{p},\;a_{o}$ are the inputs along with input reference axis, pendulum reference axis, and output reference axis; $K_{0}$ is bias; $K_{1}$ is scale factor; $K_{2}$ is secondary-order nonlinear coefficient; $K_{3}$ is third-order nonlinear coefficient; $K_{ip}$ is the coupling coefficient of input reference axis and pendulum reference axis; $K_{io}$ is the coupling coefficient of input reference axis and output reference axis; $\delta_{o}$ is misalignment angle of output axis; $\delta_{p}$ is misalignment angle of pendulum axis.

Through standardized production and installation, $K_{3}$, $\;K_{ip}$,$\;K_{io}$, $\;\delta_{o}$ and $\delta_{p}$ can be negligible in the simplified model of QFA. The error of $K_{2}$ is closely related to high acceleration, whose uncoupling effect with temperature is weak. Therefore, the simple model of QFA which only includes bias and scale factor is used to address the temperature issue.

The simple temperature model of QFA is usually used as follows [14]:

(2) $$E_{out}(T)=K_{1}(T)\cdot \left[K_{0}(T)+a_{i}\right]$$

where $E_{out}(T)$ is the output value of QFA at T°C; $K_{1}(T)$ is the scale factor at T°C; $K_{0}(T)$ is the bias at T°C; $a_{i}$ is the specific force acting along the QFA input axis.

Scale factor and bias are compensated separately through a four-point calibration method at different temperatures. Normally, the scale factor and bias are fitted through simple linear regression. The scale factor and bias are shown below:

(3) $$K_{1}(T)=\frac{E_{90^{{}^{\circ}}}(T)-E_{0^{{}^{\circ}}}(T)}{2}=\phi \cdot ∆T+K_{1}(T_{0})$$

(4) $$K_{0}(T)=\frac{E_{180^{{}^{\circ}}}(T)+E_{270^{{}^{\circ}}}(T)}{2K_{1}(T)}=\omega \cdot ∆T+K_{0}(T_{0})$$

where ϕ and ω are coefficients of scale factor and bias; $∆T=T-T_{0}$ is the difference of temperature compared with reference temperature.

The compensated output of QFA by using (3) and (4) is:

(5) $$a_{i}(T)=\frac{E_{out}(T)}{K_{1}(T)}-K_{0}(T)$$

### 2.2. Temperature Hysteresis Model of QFA

**Figure 1:**
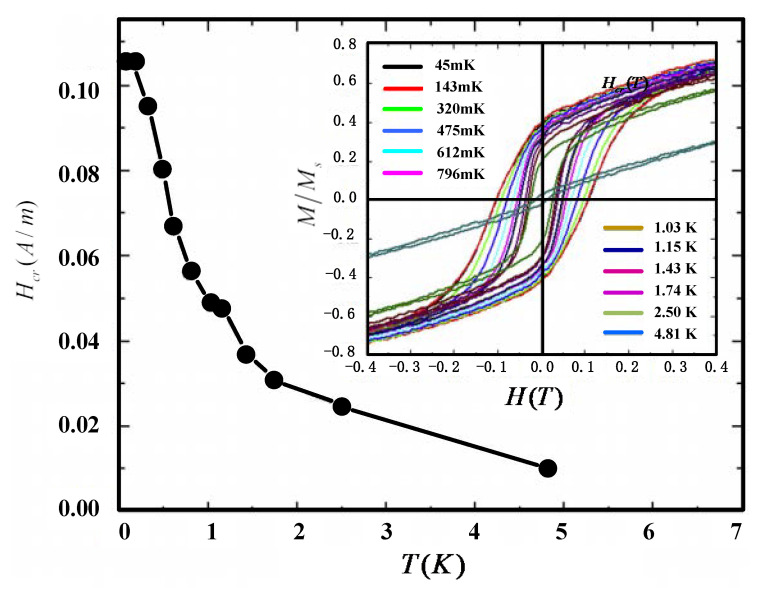
Temperature-dependence hysteresis and coercive force for permanent magnet [Ru_2_(O_2_CMe)_4_]_3_[Fe(CN)_6_]. The main panel shows that the coercive force decreases with temperature decreasing. The coercive force decreases slowly with increasing temperature, but abruptly decays in the vicinity of the transition temperature. The inset shows the magnetic hysteresis loops between 40 mK and 4.8 K. The smallest hysteresis occurs at highest temperature 4.8 K, which then grows with decreasing temperature until saturating below 143 mK.

QFA consists of the permanent magnet, quartz, ER, iron, and polyester, whose temperature characteristics vary greatly [15]. A mass of research has analyzed the temperature hysteretic behavior of the magnet, which has been proved as the main factor affecting the scale factor performance. Taking a [Ru_2_(O_2_CMe)_4_]_3_[Fe(CN)_6_] permanent magnet as an example, the temperature hysteresis at different temperatures from 40 mK to 4.8 K is shown in Figure 1 [16]. Almost every temperature-dependence permanent magnet, like Alnico and Nd_2_Fe_14_B, is temperature-dependence hysteretic and the shape of the hysteretic curve is similar to the inset of Figure 1 [17]. The Alnico permanent magnet which is used in QFA has the similar hysteresis properties as [Ru_2_(O2CMe)_4_]_3_[Fe(CN)_6_] in the temperature cycle from −195 °C to 400 °C [18,19].

The QFA scale factor is mainly determined by magnetic induction. Therefore, the temperature hysteretic behavior of magnetic is the key factor of temperature hysteresis of scale factor. Scale factor of QFA can be simply described as a second-order model:

(6) $$K_{1}(T)=q\cdot {\left(∆T\right)}^{2}+w\cdot ∆T+r$$

where q, w, and r are the temperature coefficients of the scale factor.

According to the analysis of the hysteretic phenomenon of scale factor, parameters of scale factor at a specific temperature are not only affected by ∆T, but also affected by gradient of temperature $\frac{\partial T}{\partial t}$ (in this paper, temperature gradient refers to the rate of temperature changing over time). Therefore, the scale factor is remodeled as:

(7) $$K_{1}(T)={\left(a\frac{\partial T}{dt}+b\right)}^{2}{\left(∆T\right)}^{2}+{\left(c\frac{\partial T}{dt}+d\right)}^{2}\left(∆T\right)+{\left(e\frac{\partial T}{dt}+f\right)}^{2}$$

(8) $$a\frac{\partial T}{\partial t}+b=q$$

(9) $$c\frac{\partial T}{\partial t}+d=w$$

(10) $$e\frac{\partial T}{\partial t}+f=r$$

Linear fitting of scale factor shows the magnitude of coefficients of ${\left(\frac{\partial T}{dt}\right)}^{2}∆T,\;{\left(\frac{\partial T}{dt}\right)}^{2}{\left(∆T\right)}^{2},\;and\;\frac{\partial T}{dt}{\left(∆T\right)}^{2}$ are 10^−5^ (Using the IMU data in flight experiment). Because the QFA is assembled in the middle of the INS, $\frac{\partial T}{\partial t}$ and ∆T are usually small in aircraft. Therefore, high order terms of Equation (7) are small terms.

After combining similar terms and omitting the high order small terms, Equation (7) can be simplified as below:

(11) $$K_{1}(T)=\alpha \cdot {\left(\frac{\partial T}{dt}\right)}^{2}+\beta \cdot {\left(∆T\right)}^{2}+\eta \cdot \frac{\partial T}{dt}\cdot ∆T+\varepsilon \cdot \frac{\partial T}{dt}+\mu \cdot ∆T+\gamma$$

where α,β,η,ε,μ,γ are temperature coefficients of scale factor.

Therefore, based on Equation (11), Equation (2) can be remodel as the temperature hysteresis model:

(12) $$E_{out}(T)=\left[\alpha \cdot {\left(\frac{\partial T}{dt}\right)}^{2}+\beta \cdot {\left(∆T\right)}^{2}+\eta \cdot \frac{\partial T}{dt}\cdot ∆T+\varepsilon \cdot \frac{\partial T}{dt}+\mu \cdot ∆T+\gamma \right]\cdot \left[K_{0}(T)+a_{i}\right]$$

ER is mainly used as the adhesive to bond the coil with the quartz pendulum whose form relates to bias [19]. Because of the steep temperature gradient and the wide range of temperature in aerial conditions, the thermal influence on ER is typically obvious. Therefore, the deformation of the ER possibly degrades the stability of the pendulum structure, which affects the performance of bias.

**Figure 2:**
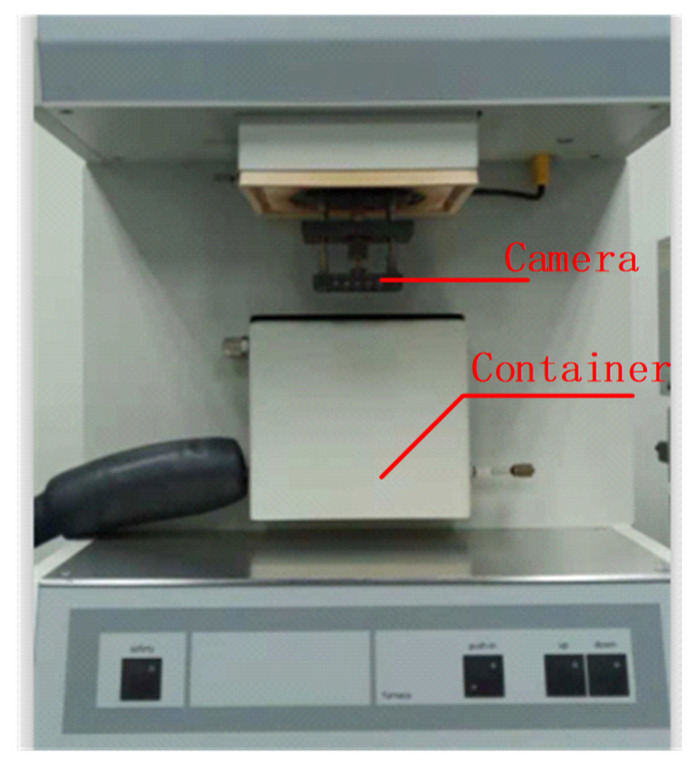
The equipment of creep experiment mainly consists of two parts, a high-resolution camera and a container. Camera can record the micro deformation of object in the container, and the container is a temperature-controlled oven which can provide precise temperature from −70 °C to +100 °C.

ER is of viscoelasticity, which means it has both elastic and viscous properties. Static viscoelasticity and dynamic viscoelasticity are two main characteristics of viscoelasticity. Static viscoelasticity responds to creep effect and hysteretic effect. Dynamic viscoelasticity responds to thermal expansion [20,21,22,23]. When ER is affected by the variation of external temperature, internal stress changes. ER stores part of the stress effect and expends the other part, which corresponds to thermal expansion. The stored stress is released when external temperature recovers, which forces the ER to restore to its original condition [24,25,26]. However, the expended stress causes creep whose deformation is irregular [27]. This phenomenon indicates that the hysteresis of bias has a strong relation with the viscoelasticity of ER. In order to figure out the influence of the static viscoelasticity on QFA, an ER temperature experiment was conducted. The equipment used is shown in Figure 2.

Three blocks of ER of same shape, weight and volume were put in three containers whose inner temperatures were set to −55 °C, 30 °C and 85 °C, respectively. Because of the setting of the camera, the measurement of deformation was in negative form. The experiment was conducted for 10 times and the creep results are shown in Table 2. When the structure of ER was relatively stable, the deformation $∆L_{v}$ corresponding to creep was recorded.

The results show that deformation caused by the creep effect related to the temperature and the size of deformation increases while temperature increases. However, deformation at the same temperature varies greatly, which means a precise creep-temperature deformation model is hard to be built.

The experiment results of deformation caused by thermal effect are shown in Table 3. $∆L_{c}$ is the severe deformation which caused by thermal effect. The variation of $∆L_{c}$ is small, which indicates the relationship between the thermal deformation and the temperature is relative stable. Therefore, thermal-temperature deformation model should exist.

**Figure 3:**
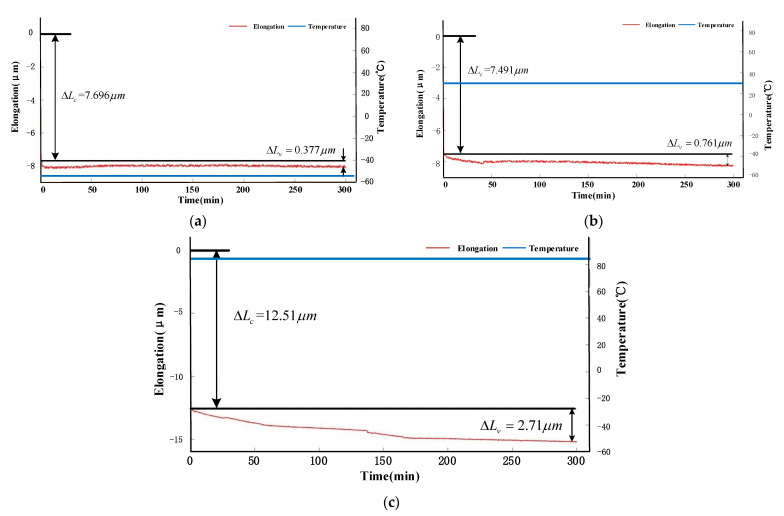
(**a**) is the elongation of ER at −55 °C; (**b**) is the elongation of ER at −35 °C; (**c**) is the elongation of ER at −85 °C. The blue curve represents temperature and the red curve represents the elongation of ER.

The result of #1 is shown in Figure 3. $∆L_{c}$ is the severe deformation whose curve is not shown in these figures.

Figure 3a shows that ER shrank 7.696 μm when it deformed severely, then it shrank slowly 0.377 μm in 300 min. Figure 3b shows that ER expanded 7.491 μm when it deformed severely, then it expanded slowly 0.761 μm in 300 min. Figure 3c shows that ER expanded 12.51 μm when it deformed severely, then it expanded slowly 2.71 μm in 300 min. The result shows temperature affects the creep, and the creep speed is proportional to temperature.

The severe deformation of ER is attributed to thermal expansion whose inner factor is dynamic viscoelasticity. The size of slow deformation and the stabilization time is constrained by temperature. This phenomenon verified that creep and hysteresis of ER are influenced by temperature, whose inner factor should be attributed to static viscoelasticity. Because creep of ER results in unexpectable pendulum deformation, $K_{0}(T)$ is probably inexplicit.

## 3. Experimental Design and Verification

### 3.1. QFA Temperature Calibration Experiment

**Figure 4:**
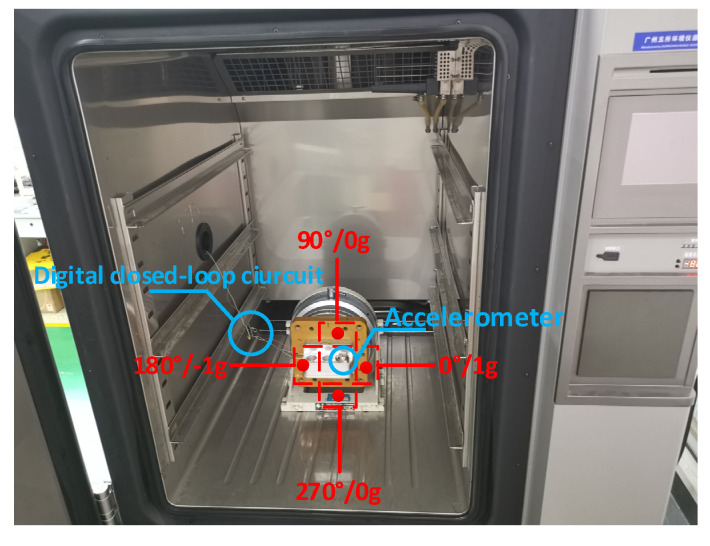
QFA assembled on an index head in temperature-controlled oven, and the index head can be controlled by oven to rotate. The inputs to QFA at 0°, 90°, 180°, 270° are 1 g, 0 g, −1 g, 0 g.

The three QFAs are JB-KT8 #1, JB-KT8 #2 and JB-KT8 #3 manufactured by Kaituo Precise Instrument Manufacturing Co., Ltd. (Baoding, China). The three QFAs are assembled in an index head and located in a high-accurate temperature-controlled oven which can provide precise temperature ranging from −75 °C to 120 °C. The temperature accuracy is better than ±0.2 °C. Temperature slew rate is ±1 °C/min to ±5 °C/min. The test equipment is shown in Figure 4.

Aerial inertial navigation systems are usually assembled in the central of the aircraft where the variation of temperature is relative stable compared to the temperature near the engine. According to the usual flight data, the gradient of temperature is about 0.2 °C/min in flight. Temperature gradient 1 °C/min can cover all temperature gradient of less than 1 °C/min. Therefore, the use of a 1 °C/min temperature gradient in QFA calibration experiments is reasonable for an aerial environment. The steps of the four-points calibration experiment are as follows:

Step 1: Fix the index head to 0°, then keep the whole device at 22 °C for 120 min;
Step 2: Temperature goes down to −60 °C at the rate of −0.3 °C/min, and it lasts 3 min at each integer temperature point;
Step 3: Temperature rises to 70 °C at the rate of 0.3 °C/min, and it lasts 3 min at each integer temperature point;
Step 4: Temperature goes down to 22 °C at the rate of −0.3 °C/min, and it lasts 3 min at each integer temperature point. Record the output of QFA.
Step 5: Repeat the step 1~step 4 at 90° (0 g), 180° (−1 g), 270° (0 g), and record the outputs of QFA.
Step 6: Repeat the step 1~step 5 with the temperature rate of 0.5 °C/min, 0.7 °C/min, and 0.9 °C/min respectively, and record the outputs of QFA.

Scale factor and bias at T°C can be expressed as:

(13) $$K_{1}(T)=\frac{A_{90{}^{\circ}}(T)-A_{270{}^{\circ}}(T)}{2}$$

(14) $$K_{0}(T)=\frac{A_{0{}^{\circ}}(T)+A_{180{}^{\circ}}(T)}{2K_{1}}$$

where $A_{0^{\circ }}(T),A_{90^{\circ }}(T),A_{180^{\circ }}(T),A_{270^{\circ }}(T)$ are average outputs of QFA at each position at *T* °C.

**Figure 5:**
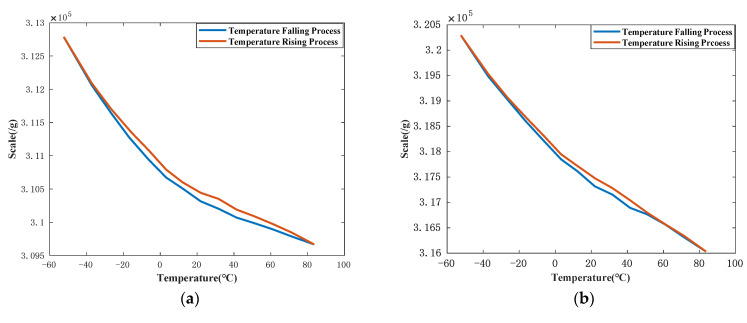

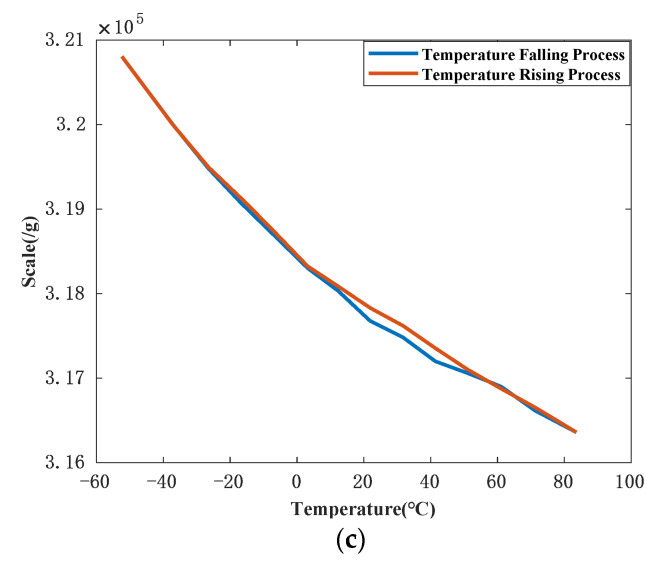
(**a**) is the scale factor of JB-KT8 #1 at different temperature; (**b**) is the scale factor of JB-KT8 #2 at different temperature; (**c**) is the scale factor of JB-KT8 #3 at different temperature.

Based on Equation (13), scale factors at different temperatures can be calculated. Curves of scale factors vs. temperature are shown in Figure 5. In these figures, the upper curves represent the temperature-falling process, and the lower curve represents the temperature-rising process. Temperature sensors (DS18B20, Maxim Integrated, San Jose, CA, USA) were installed on the surface of QFA. Therefore, the readout of the DS18B20 was not exactly the same as it controlled by the temperature oven. The vertical axes of these three figures are numbers of pulse per gravitational acceleration. The results show that the scale factor of QFA is severely affected by the temperature. The scale factor decreases while temperature rising and the scale factor increases while temperature falling. The scale factor of JB-KT8 #1 changes about 10,010 ppm from about −54 °C to 81 °C and the temperature sensitivity of scale factor is 74 ppm/°C. The scale factor of JB-KT8 #2 changes about 13,600 ppm from about −52 °C to 82 °C and the temperature sensitivity of scale factor is 104 ppm/°C. The scale factor of JB-KT8 #3 changes about 14,200 ppm from about −53 °C to 82 °C, and the temperature sensitivity of scale factor is 105 ppm/°C. (ppm means part per million which is usually used to describe the degree of change. For example, scale factor changes from X to Y, the average of scale factor is A, and the variation of temperature is T The change of scale factor normally defines as (X−Y)A×10^6 ppm. The temperature sensitivity of scale factor normally defines as (X−Y)A⋅T×10^6 ppm/°C).

The readout of temperature in this experiment is the surface temperature of QFA. In this temperature model, $\frac{\partial T}{\partial t}$ is a constant, and only ∆T is variable. Therefore, $K_{1}(T)$ can be fitted by the least squares method.

JB-KT8 #1: The expression of scale factor is shown in Equation (15):

(15) $$\begin{array}{ll} K_{1}(T)= & 273\cdot {\left(\frac{\partial T}{\partial t}\right)}^{2}+0.1008\cdot {\left(∆T\right)}^{2}\\ & -0.02409\cdot \left(\frac{\partial T}{\partial t}\right)\cdot \left(∆T\right)-32.76\cdot \left(∆T\right)+88.14\cdot \left(\frac{\partial T}{\partial t}\right)+320320 \end{array}$$

JB-KT8 #2: The expression of scale factor is shown in Equation (16):

(16) $$\begin{array}{ll} K_{1}(T)= & 102.76\cdot {\left(\frac{\partial T}{\partial t}\right)}^{2}+0.08\cdot {\left(∆T\right)}^{2}\\ & -1.4912\cdot \left(\frac{\partial T}{\partial t}\right)\cdot \left(∆T\right)-49.81\cdot \left(∆T\right)+21.01\cdot \left(\frac{\partial T}{\partial t}\right)+308000 \end{array}$$

JB-KT8 #3: The expression of scale factor is shown in Equation (17):

(17) $$\begin{array}{ll} K_{1}(T)= & 49.12\cdot {\left(\frac{\partial T}{\partial t}\right)}^{2}+0.2385\cdot {\left(∆T\right)}^{2}\\ & -0.0972\cdot \left(\frac{\partial T}{\partial t}\right)\cdot \left(∆T\right)-41.14\cdot \left(∆T\right)+48.53\cdot \left(\frac{\partial T}{\partial t}\right)+328000 \end{array}$$

**Figure 6:**
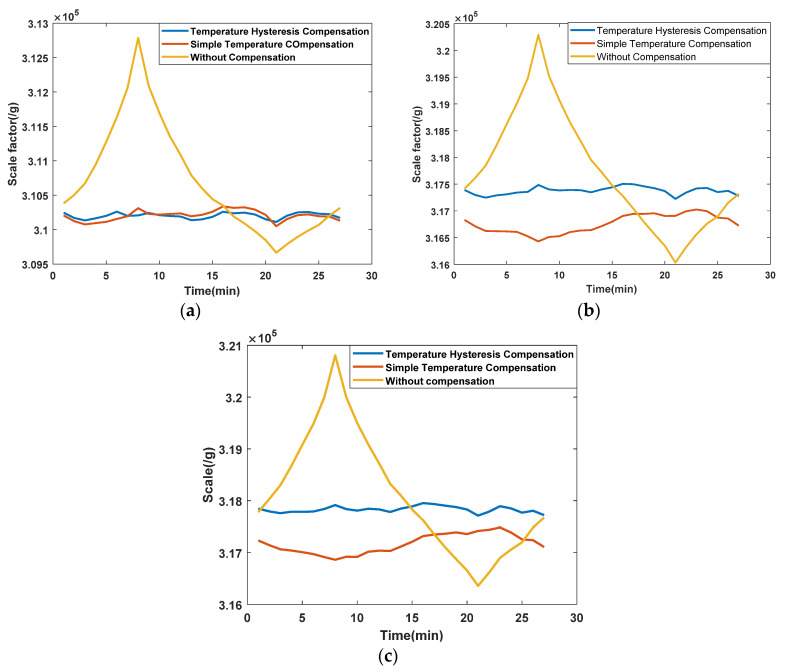
(**a**) is compensation of scale factor of JB-KT8 #1; (**b**) is Compensation of scale factor of JB-KT8 #2; (**c**) is Compensation of scale factor of JB-KT8 #3.

Based on the temperature hysteresis model, the scale factors of the three QFAs were compensated. One of the best results of compensated scale factor vs. time are shown in Figure 6, and the temperature sensitivity of the compensated scale factor is shown in Table 4.

The compensation results in Table 4 indicate that the performance of the proposed temperature compensation model improves at least an order of magnitude compared to the simple model of scale factor. With simple temperature compensation, the temperature sensitivity of scale factor is about 10 ppm/°C. The proposed temperature compensation model can improve temperature sensitivity of scale factor better than 5 ppm/°C.

**Figure 7:**
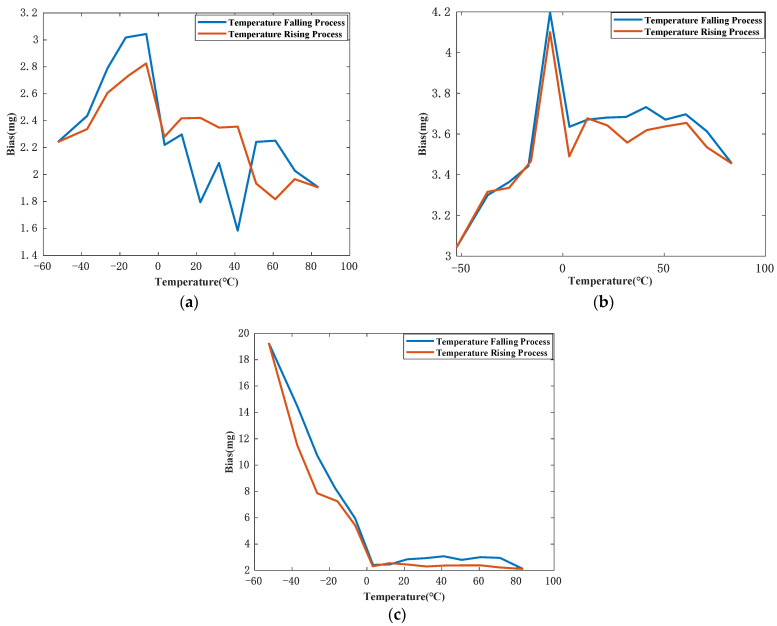
(**a**) is the bias of JB-KT8 #1 at different temperature; (**b**) is the bias of JB-KT8 #2 at different temperature; (**c**) is the bias of JB-KT8 #3 at different temperature.

Curves of bias vs. temperature are shown in Figure 7. These three figures demonstrate that there is no explicit relation between $K_{0}$ and temperature. Therefore, in order to improve the accuracy of temperature compensation of bias, a simple piecewise function is applied. Because the bias-hysteresis model is inexplicit, the detailed model and compensation result for bias are not shown in this paper.

Before the flight experiment, laboratory test of INS was implemented. The bias stability of gyroscope is 0.01°/h, and the bias stability of QFA (JB-KT8 #1, JB-KT8 #2 and JB-KT8 #3) is 50 μg in stable environment. The performance of tested INS is shown in Table 5. The same INS was used in the flight experiment.

The comparative experiments were conducted in the temperature-controlled oven to verify the efficiency of the temperature hysteresis compensation method. In order to realize the high precision temperature compensation of INS, the temperature compensated output of QFA at different temperature was collected to refine the temperature hysteretic model in system-level.

**Figure 8:**
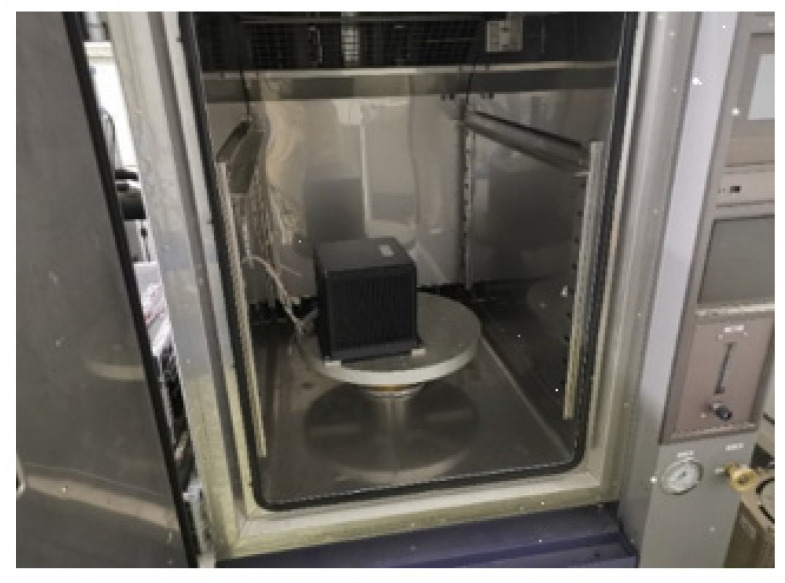
INS in temperature-controlled oven.

The comparative experiment was employed the simple temperature compensation method (stated in Section 2.1). The testing IMU was placed in the temperature-controlled oven as shown in Figure 8. The temperature is set the same as it is in real flight conditions.

**Figure 9:**
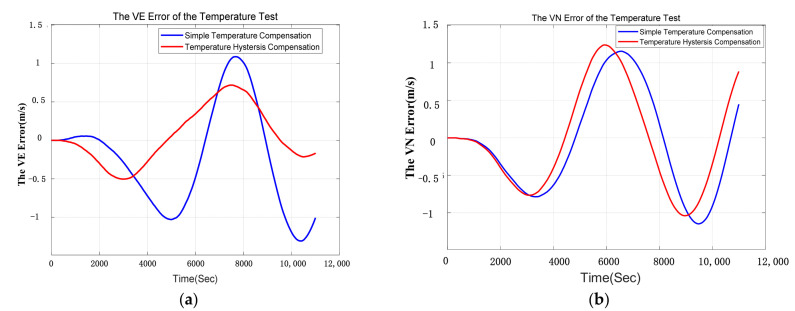
(**a**) is the comparison of east velocity error in laboratory test; (**b**) is the comparison of north velocity error in laboratory test.

The laboratory experiments of INS are conducted for 10 times and one of the best results of the comparative experiments is shown in Figure 9 and Table 6. The blue curve indicates the navigation error compensated by simple compensation and the red curve indicates the navigation error compensated by temperature hysteresis compensation. It can be concluded that the east velocity error (VE error) and the longitude error are restrained obviously, and the north velocity error (VN error) and the latitude error are also slightly reduced.

**Figure 10:**
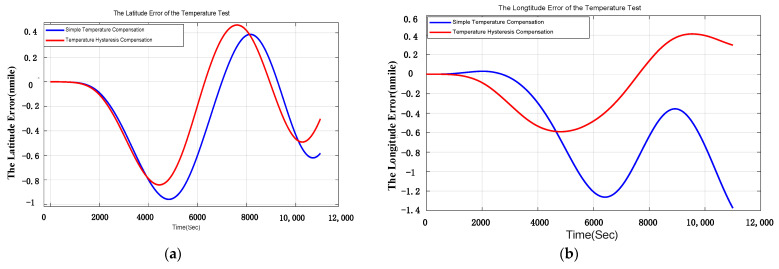
(**a**) is the comparison of longitude error in laboratory test; (**b**) is the comparison of latitude error in laboratory test.

The comparison of east velocity error and the north velocity error in laboratory experiment are shown in Table 6. The errors of east velocity and north velocity are restrained by 24.5% and 11.5%. The position error is shown in Figure 10. It is obvious that the position error decreases after temperature hysteresis compensation.

### 3.2. Temperature Hysteresis Compensation of QFA in Flight Experiment

**Figure 11:**
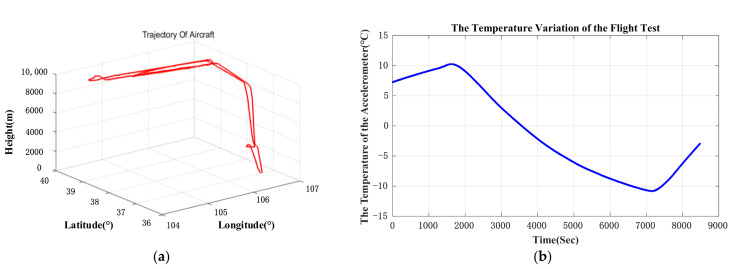
(**a**) is flight path of aircraft; (**b**) is temperature of IMU during the flight.

A flight test was conducted with the GNSS/INS in November 2019. The INS was installed outside the plane. The flight altitude was about 10,000 m, and the flight lasted for around 2.3 h. The GPS was used as a reference to evaluate the performance of the INS. The outputs of INS, including angular rate, acceleration, position, and velocity, are synchronized with the outputs of GPS. The flight path of the aircraft is shown in Figure 11a. There is no temperature control system for INS and the temperature of INS is measured by a temperature sensor assembled inside of IMU. The INS used in the flight experiment is composed of 3 parts: IMU, navigation computer, and power module. IMU is set in a cabin which is isolated from other parts. We used the temperature readout of IMU as the temperature of INS. IMU consists of three accelerometers and three gyroscopes which are assembled on an aluminum alloy fixture whose temperature distributes uniformly. Therefore, the temperature of INS is equivalent to the surface temperature of aluminum alloy fixture whose temperature is almost the same as the surface temperature of the accelerometer. The temperature of IMU is shown in Figure 11b. The flight phase was divided into three parts:

Start-up and Climbing: It took about 30 min to climb to 10,000 m. The temperature of the INS rose at the beginning period because of the heat generated by the circuits of INS.

Cruise: The aerial vehicle cruised at the altitude of 10,000 m for 1.5 h. The temperature of the INS fell constantly to −11 °C.

Landing: This lasted about 20 min, and the temperature of the INS was rising as the altitude decreased.

**Figure 12:**
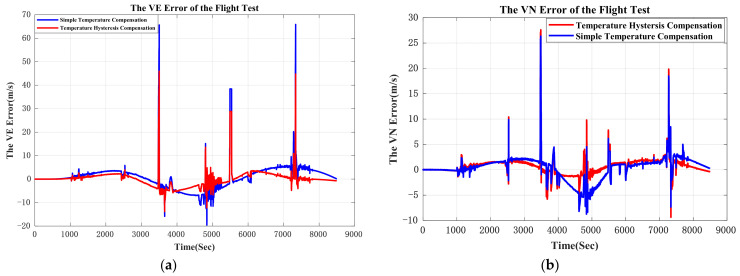
(**a**) is east velocity error; (**b**) is north velocity error. Because the experiment GNSS/INS was assembled inside the cabin and the GPS antenna cannot be installed outside of the cabin, GPS signal was lost when roll angle of the plane was bigger than 30°. GPS signal blocked by structure of cabin at big roll angle may be responsible for GPS loss. The vertexes in the graph are caused by the GPS signal loss.

The original output of the GNSS/INS was processed offline. The output of the QFA was compensated by the proposed method and simple temperature compensation method respectively. Furthermore, these two groups of processed data were applied to the same pure inertial navigation algorithm to evaluate the compensation effect. The east velocity error and north velocity error are shown in Figure 12.

The comparison of east velocity error and the north velocity error are shown in Table 7. The errors of east velocity and north velocity are restrained by 19.9% and 15.0%.

**Figure 13:**
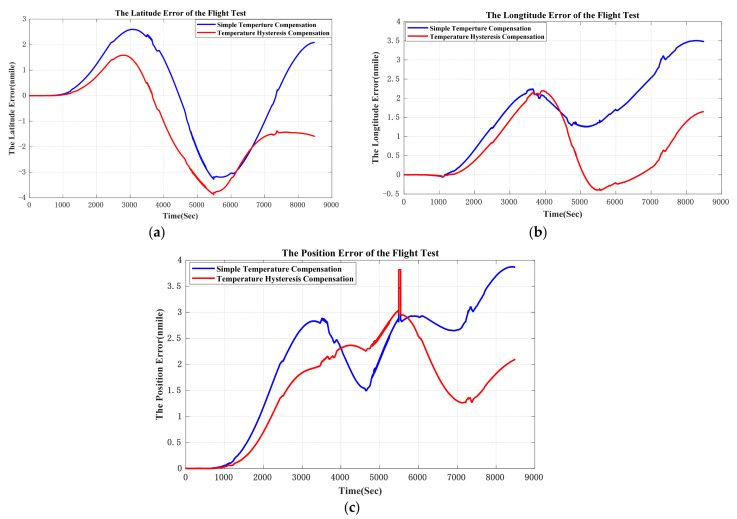
(**a**) is latitude error of the flight test; (**b**) is longitude error of the flight test; (**b**) is position error of the flight test. In (**b**) and (**c**), because the GPS signal was smoothened by simple linear regression, small mutations in position calculation appeared when GPS signal loss.

Navigation errors are shown in Figure 13. In order to fix the problem of GPS signal loss, GPS data was smoothened through simple linear regression when conducting an off-line navigation solution. The temperature hysteresis compensation result in flight condition shows that the position error (CEP) is restrained from 1.54 nmile/h to 1.29 nmile/h, which means the accuracy of navigation improves at least by 16.2%. In Figure 13, the difference between position errors compensated by two models at early stage is small. This is because the variation of temperature and the gradient of temperature is small, and the simple temperature compensation model works well at this temperature condition. After 6000 s, plane cruised at 10,000 m and then landed. In this period, the temperature varied dramatic, which indicates that the performance of proposed temperature model is better than conventional temperature model.

## 4. Discussion

According to the analysis above, temperature variation influences the performance of QFA greatly, which degrades the precision of navigation in aerial conditions. Conventional temperature compensation for accelerometers could improve the performance of the INS in aerial conditions. However, the performance of conventional temperature compensation model of QFA cannot meet the demand. Therefore, further research on temperature issues is vital to improve the temperature performance of QFA in severe environments.

In this paper, we propose a temperature hysteresis compensation method for QFA. Through the analysis of the characteristic of ER and magnet, we derived the basic cause of the temperature hysteresis phenomenon which relates to both temperature and the temperature gradient. Based on the analysis above, a rational temperature model is built for accelerometers. Proper QFA temperature compensation experiments were designed to refine the model. The comparative experiments with a simple temperature compensation method in the laboratory indicate that the efficiency of the proposed compensation method is better than the simple one. The result of pure inertial navigation in the flight experiment shows that the navigation accuracy (CEP) improves by 16.2%, from 1.54 nmile/h to 1.29 nmile/h, after temperature hysteresis compensation.

Beyond the proposed temperature compensation method, our future work will focus on optimizing the experiment method and building precise bias-temperature model. The bias has a hysteretic phenomenon, but the bias-hysteresis model remains inexplicit. Using a piecewise function for bias compensation is effective while it still needs to be optimized. Temperature compensation for QFA is not the only one way to improve its performance. The more effective approach is to optimize QFA itself. For example, the differential structure in the QFA is the focus of our future research. Besides, it should be taken into consideration that the parameters of the QFA temperature model in the INS may be different from the parameters in the single QFA, so precise temperature compensation in the INS level need to be conducted.

## Figures and Tables

**Table 1:** Main performance characteristics of QA3000 and GJN06.

	QA3000-030	GJN096-D
Input range [g]	±60	±70
Bias repeatability [μg]	<40	<60
Bias temperature sensitivity [μg/°C]	15	50
Scale factor repeatability [ppm]	<80	<80
Scale factor temperature sensitivity [ppm/°C]	120	50
Operating temperature [°C]	−28~+78	−48~+80
Shock [g]	100	200
Resolution/Threshold [μg]	<1	<5
Bandwidth [Hz]	>300	<2000

Data are from their respective product descriptions.

**Table 2:** Results of creep deformation of ER.

	−55 °C	30 °C	85 °C
$∆L_{v}{}_{1}$ [μm]	−0.377	−0.761	−2.710
$∆L_{v2}$ [μm]	−0.258	−0.889	−3.804
$∆L_{v3}$ [μm]	−0.476	−0.575	−2.229
$∆L_{v4}$ [μm]	−0.477	−0.659	−2.401
$∆L_{v5}$ [μm]	−0.393	−0.685	−3.373
$∆L_{v6}$ [μm]	−0.482	−0.982	−2.898
$∆L_{v7}$ [μm]	−0.579	−0.676	−4.312
$∆L_{v8}$ [μm]	−0.252	−0.864	−3.915
$∆L_{v9}$ [μm]	−0.572	−0.961	−2.209
$∆L_{v10}$ [μm]	−0.281	−0.782	−2.921
Variation	84.88%	42.44%	77.60%

Taking #1 result as benchmark, $Variation=(∆L_{v_{\max}}-∆L_{v_{\min}})/∆L_{v_{1}}\cdot 100\%$.

**Table 3:** Results of thermal deformation of ER.

	−55 °C	30 °C	85 °C
$∆L_{c}{}_{1}$ [μm]	−7.696	−7.491	−12.51
$∆L_{c2}$ [μm]	−7.674	−7.502	−12.56
$∆L_{c3}$ [μm]	−7.712	−7.489	−13.05
$∆L_{c4}$ [μm]	−7.703	−7.482	−12.39
$∆L_{c5}$ [μm]	−7.671	−7.409	−12.18
$∆L_{c6}$ [μm]	−7.692	−7.511	−12.86
$∆L_{c7}$ [μm]	−7.710	−7.488	−12.32
$∆L_{c8}$ [μm]	−7.685	−7.492	−12.54
$∆L_{c9}$ [μm]	−7.693	−7.508	−12.77
$∆L_{c10}$ [μm]	−7.749	−7.491	−12.59
Variation	1.01%	1.32%	5.8%

Taking #1 result as benchmark, $Variation=(∆L_{c_{\max}}-∆L_{c_{\min}})/∆L_{c_{1}}\cdot 100\%$.

**Table 4:** Compensation of temperature sensitivity of scale factors.

	Without Compensation (ppm/°C)	Simple Method (ppm/°C)	Proposed Method (ppm/°C)
JB-KT8 #1	74.14	12.78	2.48
JB-KT8 #2	104.22	14.14	4.23
JB-KT8 #3	105.79	14.46	5.19

**Table 5:** Performance of the tested INS.

	Performance	Quantity
ACC	Bias Stability	50 μg
	Bias Repeatability (one month)	50 μg
	Scale factor-Factor Linearity	30 ppm
	Scale factor-Factor Repeatability (one month)	30 ppm
GYRO	Bias Stability	0.01°/h
	Scale factor Repeatability (one month)	0.01°/h
	Scale factor-Factor Linearity	20 ppm
	Scale factor-Factor Repeatability (one month)	20 ppm

**Table 6:** Comparison of velocity error in laboratory experiment.

	East Velocity	North Velocity
Simple compensated (RMS) m/s	0.98	1.04
Compensated (RMS) m/s	0.74	0.92

**Table 7:** Comparison of velocity error in flight experiment.

	East Velocity	North Velocity
Simple compensated (RMS) m/s	1.36	1.4
Compensated (RMS) m/s	1.09	1.19

## Data Availability

Data sharing not applicable.
